# Arsenious Acid in Dentistry

**Published:** 1891-07

**Authors:** 


### Editorial.

### ARSENIOUS ACID IN DENTISTRY.

The fact that the world of thought moves in circles is nowhere more apparent than in dentistry. This is, perhaps, inevitable, from the fact that each generation brings with it men cultured, it may be, in the scientific thought of the time, but not familiar with the past history of their profession, as they should be, nor conversant, as they certainly ought to be, with the treatment of pathological conditions in past epochs.

The extraordinary rush of apparent intelligence into what may be called the startling in therapeutics is without reason except that individuals may regard this as an open door to fame or notoriety; the difference between these two states may not be clear to some minds, and to them the terms are possibly interchangeable.

When Spooner, in 1836, introduced arsenic for the devitalization of pulps, he probably had no idea that fifty-four years thereafter it would be recommended for filling root-canals; but such has recently been done, and the matter gravely discussed by a learned society.[1]

Arsenious acid, from its peculiar action, is fitted for the purpose of devitalizing pulps as no other known agent. It is not necessary to call the attention of our readers to any extent to its peculiar properties. These are well understood, and were it not for the fact that some seem to forget that in using it they are not only dealing with an escharotic, but one of the most powerful and insidious of poisons, it would not be necessary to allude to it here.

While there is still much to learn in regard to the action of this agent in its local, general, and cerebral effects, it is well understood that it does not destroy the tissue as some other escharotics, but the destruction of life is through a more complicated process, and has no immediate effect upon the continuity of the pulp. The action of arsenious acid is to first render the sensory nerves torpid, for Sklarek, quoted by Ringer, says, "Arsenic, therefore, paralyzes first sensation and reflex action and some time afterwards voluntary power." Ringer further writes: "My own experiments, conducted with Dr. Murrell, confirm this statement; but they show also that arsenious acid is a paralyzer of the motor and sensory nerves and of the muscles."

---

[1] Mississippi Valley Association of Dental Surgeons, March, 1891.

Probably no class of professional men have had this drug more closely under observation than dentists. For over fifty years it has been their one agent for the destruction of pulps, and when properly used is always reliable. Hence those who have observed closely have long since arrived at the conclusions quoted, and that the paralysis observed was a gradual process requiring a definite time for its completion, and its extent was only limited by the amount of arsenic applied. The torpidity of the sensory nerves of the pulp means a retardation of the blood-supply, and eventually death.

The action of this agent shows also a clearly-defined progress, and an examination of a removed pulp will exhibit a distinct line of demarcation between the affected and the non-affected portion, and this is of deep interest in a macroscopic as well as in a microscopic sense. It will have been noticed, in practice, that the antiseptic property of arsenious acid is of no value. This, in the discussion alluded to, was dwelt upon as the main reason for its use. While the agent fails to break up the tissue, it leaves the pulp and, consequently, all its connections in a condition to rapidly decompose, which takes place in a varying period of from ten days to two weeks.

The evidences are ample to demonstrate that the use of arsenious acid should be confined to that purpose which led to its introduction, and that even here it will fail and be productive of serious results in the hands of the careless or ignorant.

In the annual address alluded to the following formula was offered for "root-filling:"

℞ Arsenious acid, gr. ii;
Precipitated chalk, ʒi;
Glycerin, q.s.
Sig.—To be made into a paste.

He says, "The dose of the arsenic is from $\frac{1}{20}$ to $\frac{1}{40}$ of a grain. The $\frac{1}{40}$ of a grain is probably all that is applied in this use of the drug, and this is *not in contact with soft tissue.* Even should it be forced through the foramen *its effect would only be beneficial.*" (Italics ours.) It is not necessary to enter into any lengthy discussion of this quotation, as it probably carries with it its own antidote.

His mode of filling the canals is as follows: He takes "one-half grain of the mixture, equal in size to half a grain of wheat, mix with a single drop of water on a slab. Introduce into the canal, which should first be repeatedly washed with alcohol and thoroughly dried. Fill the canal perfectly with the compound. Then dry again, and in the majority of cases fill the tooth at once."

The supposition that there are no avenues for the agent to reach the pericementum is a fallacious one. Not only is the foramen present as an outlet, but it has been demonstrated that not infrequently other openings are found to expose a greater surface to the irritant. It has further never been proven that a continuous chain of canals do not exist, connecting the tubuli with the pericementum. On the contrary, there is much evidence to show that solutions can be transmitted from the pulp-canal to the periphery of the tooth.

Those who have had much experience appreciate the difficulty in preventing that much-to-be-dreaded pericementitis from arsenious acid. And so far from its being true that, "even if it did pass it could do no harm," it is certain to arouse periosteal inflammation of a serious character. If it fails to affect this, it is simply because the amount has been too minute to produce results beyond a limited area of tissue.

If this agent destroys the life of the pulp, in the manner previously outlined, it will have a similar effect on all tissues. This is well understood, and the sloughing of cervical margins is an apt illustration, and demonstrates what takes place when the pericementum is saturated with the solution. The result is death of this important organ, necrosis of the cemental tissue, and necessarily destruction of the corresponding alveolar plate. That any other view should be accepted is strange; and it is, further, a matter of surprise that any organization should seriously consider the subject, or conceive, as one present expressed it, "There may be more in this method than we are now able to conceive after all."

In the May number of the Journal is a paper read before one of our own most prominent societies, in which arsenious acid and cocaine are recommended for sensitive dentine, although the writer acknowledges that "in private practice it is not desirable to try it as an obtunder, except in certain teeth like bicuspids, or others that are to be removed for regulating, for it might endanger the vitality of the pulp." While this is a saving clause, it would seem that the whole process should then and there have been denounced.

This mode of treating the hyperæsthetic conditions of dentine with arsenious acid has been repudiated by the dental profession for many years. A certain very prominent operator in this city (Philadelphia) applied it for this purpose more than a quarter of a century ago, and took one step in advance of the writer of the paper, for he regarded it possible to use it as an aid in the extraction of teeth.

It is only recently that several of the dental journals, including the International, felt called upon to expose a preparation that had deceived some of the most intelligent dentists, and we are certain to have a flood of nostrums of a similar character if those who are the leaders of thought are not constantly guarding the best interests of their profession.

Dentistry has suffered enough reproach from the careless use of this drug, and no one having its welfare at heart dare pass by any reckless application of it. It has only one legitimate place in our work, and that is the one proposed by Spooner.

